# Effect of Probiotics and Multi-Component Feed Additives on Microbiota, Gut Barrier and Immune Responses in Broiler Chickens During Subclinical Necrotic Enteritis

**DOI:** 10.3389/fvets.2020.572142

**Published:** 2020-11-26

**Authors:** Nima K. Emami, Ali Calik, Mallory B. White, Emily A. Kimminau, Rami A. Dalloul

**Affiliations:** ^1^Avian Immunobiology Laboratory, Department of Animal and Poultry Sciences, Virginia Tech, Blacksburg, VA, United States; ^2^Department of Animal Nutrition & Nutritional Diseases, Faculty of Veterinary Medicine, Ankara University, Ankara, Turkey; ^3^Huvepharma Inc., Peachtree City, GA, United States; ^4^Department of Poultry Science, University of Georgia, Athens, GA, United States

**Keywords:** necrotic enteritis, performance, immune response, gut microbiota, tight junction, lesion score

## Abstract

The withdrawal of antibiotic growth promoters from poultry feed has increased the risk of necrotic enteritis (NE) outbreaks. This study examined the effects of a probiotic (PROB) or probiotic/prebiotic/essential oil supplement (PPEO) during a subclinical NE challenge. On day (d) of hatch, 960 male broilers were randomized to four groups (8 pens/treatment, 30 birds/pen) including (1) negative control (NC): corn-soybean meal diet; (2) positive control (PC): NC + 20 g Virginiamycin/ton diet; (3) NC + 227 g PROB/ton diet; and (4) NC + 453 g PPEO/ton diet. One d after placement, birds were challenged by a coccidia vaccine to induce NE. Feed intake and body weights were measured on d 8 (NE onset) and end of each feeding period. On d 8, the small intestines of three birds/pen were examined for NE lesions. Jejunum samples and ileal mucosal scrapings from one bird/pen were respectively collected to measure mRNA abundance (d 8 and d 14) and profile the microbiota (d 8 and d 42). Data were analyzed in JMP or QIIME 2 and significance between treatments identified by LSD (*P* < 0.05). PROB and PPEO had significantly lower mortality (d 0–14) and NE lesion scores compared to NC. Feed conversion ratio was significantly lower in PC, PROB, and PPEO, while average daily gain was higher in PPEO and PC groups compared to NC from d 0–42. On d 8 and d 14, mRNA abundance of claudin-3 was higher in PPEO compared to NC. On d 14, compared to NC, mRNA abundance of sIgA and PGC-1α in PROB and PPEO were lower and higher, respectively. Compared to NC, PPEO increased mTOR abundance on d 14. On d 8, relative abundance of *Clostridium sensu stricto 1, Ruminiclostridium9, Prevotellaceae, Prevotellaceae* UCG-014, ASF356, and *Muribaculaceae* was higher in NC compared to PPEO and PROB, while *Lactobacillus* was lower in NC. *Escherichia-Shigella* had higher abundance in PC compared to PPEO and PROB. Collectively, these data indicate that during a subclinical naturally occurring NE, supplementation of PROB or PPEO supports performance and reduces intestinal lesions, potentially through modifying tight junction proteins, gut microbiota, immune responses, and cell metabolism.

## Introduction

Necrotic enteritis (NE) is a significant enteric disease in poultry with considerable economic effect on profitability. The annual financial losses to the poultry industry worldwide are estimated at $6 billion (1). NE presents a unique challenge, being a complex enteric disease that often leads to either clinical (acute) or subclinical (chronic) form (2, 3). The latter typically results in poor performance (reduced feed intake, weight gain, and eventually higher feed conversion ratio) with low mortality rates and represents the greatest economic impact on poultry production (4, 5). Predisposing factors such as *Eimeria* and fishmeal would lead to disturbances in the microbiota providing a favorable ecological environment and nutrients that allow the proliferation of *Clostridium perfringens*, thus subclinical NE occurrence (5, 6).

Gut barrier function is an important factor for the optimum health and performance of broiler chickens. Maintenance of gut integrity and function is a highly energy demanding process, thus energy deficits due to alteration in the intestinal metabolism and mitochondrial functionality may cause intestinal damage and injury (7). In addition, during infection and inflammation (e.g., NE), cell proliferation in the intestine accelerates to replace damaged enterocytes (8). This epithelial cell renewal along the crypt-villus axis is regulated by the mechanistic target of rapamycin (mTOR) signaling pathway. This may partly be due to its ability to affect the antioxidant capacity and protein synthesis of intestinal epithelial cells (9, 10). In many intestinal diseases, cell loss from the villus exceeds the regenerative capacity of the crypts (11) and shedding of epithelial cells may cause transient gaps or microerosions in the epithelial barrier resulting in increased intestinal permeability (11). Tight junctions (TJ) are protein complexes that seal the paracellular space between adjacent cells in the gut, maintaining epithelial barrier function (12). However, these barriers have a dynamic structure that remodel in response to signals from the cells or external stimuli such as nutrients, and pathogenic and commensal bacteria (13). *C. perfringens*, the pathogen responsible for NE, produces enterotoxins that bind to TJ proteins, mainly claudins (CLDN)-3 and -4 (14, 15), which eventually leads to pore formation, an increase in paracellular permeability, and cytotoxicity (15, 16). In addition to being barriers, TJ contain essential components of the signaling networks that guide diverse cell behaviors and functions and regulate epithelial proliferation and differentiation (17). As a result, TJ signaling plays an important role in general cell responses to stress. Zonula occluden (ZO) proteins are linked to the maintenance of junctional integrity in response to osmotic stress or changes in cell shape during morphogenesis in non-avian models including zebrafish and *Caenorhabditis elegans* (18). Non-avian studies evidenced relationships between immune responses and gut barrier function. As an example, interleukin (IL)-17 and IL-22 are tissue-signaling cytokines that favor protection and regeneration of cells in barrier organs such as the gastrointestinal tract (19). Cytokines could affect TJ structure and function, thus mediating paracellular permeability and exposure of tissues to luminal antigens in the gastrointestinal tract (12, 20).

One of the additive groups that has been tested for their efficacy as alternatives to antibiotic growth promoters are probiotics (21). Supplementation of probiotics promotes gut health and performance by reducing the number of pathogens in the gastrointestinal tract through competitive exclusion or production of antimicrobial peptides, thus improving intestinal maturation and integrity and modifying immune responses (22–25). Despite several reports on the effect of probiotics, less attention has been paid to the effect of multi-component additives or comparison of these additives with probiotics as alternatives to antibiotic growth promoters especially during NE challenge. In a recent study, supplementation of a multi-component additive had no effect on NE lesions in the small intestine of broiler chickens, but improved FCR during the starter and grower periods (26).

Therefore, this study was conducted in order to evaluate the effects of a probiotic or a multi-component additive on performance, pathology, RNA expression of TJ proteins, immune responses, and cell energy metabolism-related genes, as well as ileal mucosa microbial profile during a naturally occurring NE challenge model.

## Materials and Methods

### Birds, Diets, and Management

This project was approved and conducted under the guidelines of the Virginia Tech Institutional Animal Care and Use Committee (IACUC # 18-136).

A total of 960 one-day-old Cobb 500 male broiler chickens were acquired from a local hatchery. Prior to placement, birds were weighed in groups of 30 each and allocated to one of 32 pens with dimensions of 1.2 × 1.2 m for the first 14 days and 1.2 × 2.4 m from d 15 to 42. Birds were assigned to the four dietary treatments (8 pens/treatment) as follows:

1. Negative Control (**NC**): corn-soybean meal basal diet
2. Positive control (**PC**): NC + 20 g Virginiamycin/ton diet (453 g Stafac^®^20/ton)
3. Probiotic (**PROB**): NC + PROB (227 g/ton diet from 0 to 42 days of age)
4. Probiotic/prebiotic/essential oils supplement (**PPEO**): NC + PPEO (453 g/ton diet from 0 to 42 days of age).

The PROB consisted of *Bacillus licheniformis* spores (3.2 × 10^9^ CFU/g) while the PPEO supplement contained *B*. *licheniformis* spores (3.2 × 10^9^ CFU/g), 276 ppm mannan-oligosaccharides and β-glucans (1,3 and 1,6)/kg, and 8,000 ppm capsaicin (from chili peppers) and curcuma (from turmeric)/kg. Composition of the basal diet is detailed in Table 1. One large batch of the basal diet was mixed, and the amount needed for each treatment separated to which the appropriate amounts of supplements were added according to treatments. The mash diets were subsequently crumbled (starter period) or pelleted (grower and finisher periods).

**Table 1:** Composition of basal diets (as fed basis, %)^a^.

	**Period (days)**		
**Ingredients (%)**	**Starter (0–14) Crumble**	**Grower (15–28) Pellet**	**Finisher (29–42) Pellet**
Corn (7.81% CP)	59.53	64.12	65.70
Soybean meal (48% CP)	33.5	28.80	26.86
Soybean oil (9,000 kcal/kg)	2.18	2.60	3.50
Dicalcium phosphate (18.5% P, 22% Ca)	2.05	1.92	1.70
Calcium carbonate (37% calcium)	1.11	1.00	0.90
Sodium chloride	0.30	0.30	0.30
Sodium bicarbonate	0.07	0.07	0.05
DL-Methionine (990 g/kg)^b^	0.38	0.34	0.29
L-Lysine HCl (788 g L-Lysine/kg)^c^	0.37	0.35	0.24
L-Threonine (985 g/kg)^d^	0.15	0.14	0.10
Vitamin/Trace Mineral Premix^e^	0.36	0.36	0.36
**Calculated analysis (% unless specified)**			
ME (kcal/kg)	3,007	3,087	3,168
Crude protein	21.81	19.90	18.94
Total phosphorus	0.76	0.71	0.66
Available phosphorus	0.45	0.42	0.38
Calcium	0.90	0.84	0.76
Chloride	0.33	0.33	0.29
Sodium	0.16	0.16	0.15
Potassium	0.85	0.77	0.73
Methionine	0.67	0.61	0.55
Methionine + Cysteine	0.98	0.89	0.82
Lysine	1.32	1.19	1.05
Threonine	0.86	0.78	0.71
Linoleic acid	1.44	1.52	1.55
Dietary cation-anion balance	194	174	170

a *Treatments include: NC (negative control): corn-soybean meal basal diet; PC (positive control): NC + 20 g virginiamycin (453 g Stafac^®^20)/ton diet; PROB (Probiotic): NC + B. licheniformis spores at the level of 227 g/ton diet; PPEO (probiotic/prebiotic/essential oil supplement): NC + B. licheniformis spores, mannan-oligosaccharides and β-glucans (1,3 and 1,6), capsaicin from chili peppers and curcuma from turmeric. These additives were added on top to the basal diet to provide the three experimental diets besides the NC diet in every feeding period*.

b *Rhodimet^®^ NP9, ADISSEO, GA, USA*.

c *L-Lysine HCl, AJINOMOTO HEARTLAND, INC. Eddyville, IA, USA*.

d *FENCHEM Ingredient Technology, Nanjing, China*.

e *Vitamins supplied per kg diet: retinol 3.33 mg, cholecalciferol 0.1 mg, α-tocopherol acetate 23.4 mg, vitamin K3 1.2 mg, vitamin B1 1.6 mg, vitamin B2 9.5 mg, niacin 40 mg, pantothenic acid 9.5 mg, vitamin B6 2 mg, folic acid 1 mg, vitamin B12 0.016 mg, biotin 0.05 mg, choline 556 mg. Minerals supplied per kg diet: Mn 144 mg, Fe 72 mg, Zn 144 mg, Cu 16.2 mg, I 2.1 mg, Se 0.22 mg*.

Each pen was equipped with a bucket-type feeder and a nipple drinker line with fresh wood shavings as litter (~7.5 cm-deep). Birds had *ad libitum* access to water and feed from placement (d 0) until the end of the study (d 42). Lighting schedule was 24 h light for the first 3 days, reduced to 23 h light:1 h dark from d 4 to 7, and reduced further to 18 h light:6 h dark thereafter. An automatic ventilation system was used to control the environment, and temperature was maintained as follows: 32°C for the first 3 days, then gradually reduced ~3°C each week until it reached 23°C at the start of week 4 where it remained constant thereafter.

### Necrotic Enteritis Challenge

In order to simulate field conditions, a unique naturally occurring model developed on our research farm was applied for inducing NE. This model consists of spraying a high dose of commercial coccidiosis vaccine 24 h after bird placement, which in conjunction with the presence of *C*. *perfringens* spores in the barn environment leads to the development of a NE outbreak around 1 week post-vaccine application. For this trial, the Coccivac^®^-B52 vaccine (containing live oocysts of *E. acervulina, E*. *maxima, E*. *maxima* MFP, *E*. *mivati*, and *E*. *tenella*; Merck Animal Health) was prepared at the proper concentration in the lab, kept on ice, and applied at the farm.

### Mortality

Starting at placement, birds were monitored twice a day. For each dead bird, the date, body weight and cause of death were recorded. This procedure continued throughout the study (up to d 42) to record mortality/treatment for each period thus allowing for adjusting performance parameters against daily mortality.

### Performance

Upon arrival (d 0), birds were weighed in groups of 30 and assigned to each pen. Subsequently, birds were weighed on d 8 (7 days after the coccidiosis challenge, which was also the peak mortality) and at the end of starter (d 14), grower (d 28), and finisher (d 42) periods. Additionally, feed consumption was also recorded on a per pen basis on d 8, 14, 28, and 42. Finally, adjusted average daily gain (ADG), average daily feed intake (ADFI) and feed conversion ratio (FCR) were calculated for each period (days 0–8, 9–14, 15–28, and 29–42) and also for the cumulative experimental period (d 0–42).

### Necrotic Enteritis Lesion Scores

On d 8, three birds per pen were selected based on average body weight of the pen, euthanized by cervical dislocation, and the small intestines were examined for NE lesions and scored based on a 0–4 scale system. Each section of the small intestine, i.e., duodenum, jejunum, and ileum, was scored separately by personnel blinded to the treatments.

The lesion scoring criteria used were as follows (27):

0 = No gross lesions
1 = Thin-walled or friable
2 = Focal necrosis or ulceration
3 = Multifocal coalescing areas (large patches) of necrosis
4 = Severe extensive necrosis.

### mRNA Abundance of Tight Junction Proteins, Cytokines, and Master Regulators of Cell Metabolism

On d 8 and d 14, one bird/pen was selected, euthanized, and jejunal sections (1 cm) were excised to assess the mRNA abundance of TJ proteins, mucosal immune responses and key regulators of cell metabolism. Jejunal tissue samples were homogenized by a TissueLyser II (Qiagen) and total RNA extracted using RNeasy Mini Kit (Qiagen GmbH, Hilden, Germany) according to the manufacturer's instructions. Total RNA was quantified by spectrophotometry, and integrity evaluated by gel electrophoresis on 1.5% agarose gel in 0.5X TAE buffer. Two micrograms of total RNA were used to synthesize first-strand cDNA using the High Capacity cDNA Reverse Transcription Kit (Applied Biosystems, Carlsbad, CA, USA) according to the manufacturer's recommendation. The mRNA abundance of TJ proteins [occludin (OCLN), CLDN-1, CLDN-3, ZO-1, and ZO-2], cytokines [interferon (IFN)-γ, IL-1β, IL-10, and IL-17], secretory immunoglobulin (sIg)A, mTOR, AMP-activated protein kinase-α1 (AMPK- α1), and peroxisome proliferator-activated receptor-γ coactivator (PGC)-1α were determined by quantitative real-time PCR (7500 Fast Real-Time PCR System, Applied Biosystems) using Fast SYBR™ Green Master Mix (Applied Biosystems). Primer details are provided in Table 2. Each reaction was performed in a total volume of 20 μL in duplicate wells. Product specificity was confirmed by analysis of the melting curves generated by the 7500 software (version 2.0.3). mRNA abundance was analyzed using glyceraldehyde 3-phosphate dehydrogenase (GAPDH) as an endogenous control. Average mRNA abundance relative to GAPDH for each sample was calculated using the 2^−ΔΔCt^ method (29) with the calibrator for each gene being the average ΔCt value from the negative control group.

**Table 2:** Sequences of primer pairs used for amplification of target and reference genes.

**Gene**	**Primer sequence**	**Size**	**Acc (reference)**
Claudin-1	GTGTTCAGAGGCATCAGGTATC GTCAGGTCAAACAGAGGTACAA	107	NM_001013611.2
Claudin-3	CCCGTCCCGTTGTTGTTTTG CCCCTTCAACCTTCCCGAAA	126	NM_204202.1 (28)
Occludin	CCGTAACCCCGAGTTGGAT ATTGAGGCGGTCGTTGATG	214	NM_205128.1
Zonula occluden-1	GGAGTACGAGCAGTCAACATAC GAGGCGCACGATCTTCATAA	101	XM_413773
Zonula occluden-2	GCGTCCCATCCTGAGAAATAC CTTGTTCACTCCCTTCCTCTTC	89	NM_204918
Interferon-γ	GCTCCCGATGAACGACTTGA TGTAAGATGCTGAAGAGTTCATTCG	63	NM_205149.1
Interleukin-1β	CCCGCCTTCCGCTACA CACGAAGCACTTCTGGTTGATG	66	XM_015297469.1
Interleukin-10	CGCTGTCACCGCTTCTTCA CGTCTCCTTGATCTGCTTGATG	63	NM_001004414.2
Interleukin-17A	AGCTGGACCACAGCGTCAAC GGCGGAGGACGAGGATCT	57	NM_204460.1
sIgA^a^	GTCACCGTCACCTGGACTACA ACCGATGGTCTCCTTCACATC	192	S40610.1
PGC-1α^b^	ACGGAGTTCCAATCGC AACCCTTACAACCTTCACAA	220	NM_001001464.1
mTOR^c^	CATGTCAGGCACTGTGTCTATTCTC CTTTCGCCCTTGTTTCTTCACT	77	XM_417614.6
AMPK-α1^d^	ATCTGTCTCGCCCTCATCCT CCACTTCGCTCTTCTTACACCTT	125	NM_001039603
GAPDH^e^	CCTAGGATACACAGAGGACCAGGTT GGTGGAGGAATGGCTGTCA	64	NM_204305

*For each gene, the primer sequence for forward (F) and reverse (R) (5′-3′) primers, the amplicon size (bp) and the NCBI Accession number (Acc) used for the primer design are listed*.

a *Secretory immunoglobulin A*.

b *Peroxisome proliferator-activated receptor-γ coactivator 1α*.

c *Mechanistic target of rapamycin*.

d *AMP-activated protein kinase-α1*.

e *Glyceraldehyde 3-phosphate dehydrogenase*.

### 16S rRNA Gene Sequencing

On d 8 and d 42, one bird/pen was euthanized, the ileum excised, mucosal scrapings collected, snap-frozen in liquid nitrogen and then stored at −80°C until further analysis. Bacterial DNA was extracted from mucosal scrapings using the Qiagen QIAamp^®^ PowerFecal^®^ DNA kit (Qiagen GmbH, Hilden, Germany) based on the supplier's instructions. Quality and quantity of extracted DNA were measured using gel electrophoresis and spectrophotometer. All DNA samples were diluted to 300 ng/μl and 50 μl of each sample were sent to the Virginia Tech Biocomplexity Institute for sequencing. The V4 region of the 16S rRNA gene was amplified using the Illumina MiSeq kit V3 (600-cycle format, 25 million reads, read length 2 × 300). Earth Microbiome Project primer sets 515F (30) and 806R (31) generating 390 bp amplicons were used.

Data were analyzed using QIIME 2 software version 2019.10 (32). The reads were denoised using the DADA2 pipeline. The sequences flagged as non-chimeras were retained, sequence reads with >99% identity were clustered into a single operational taxonomic unit (OTU) and SILVA database version 132 was used to assign 16S rRNA sequence reads to OTU. To remove sequencing effort heterogeneity, samples for d 8 and d 42 were rarefied to 10,891 and 29,818 sequences, respectively. These were the lowest sequence reads within each sampling day.

Alpha diversity was measured using the Shannon index (H), which accounts for both abundance (richness) and evenness of the species present. To estimate the similarity of microbial community structure between groups (β-diversity), principal coordinate analysis (PCoA) based on weighted UniFrac distance (bray-curtis) were performed. To assess the association between microbial community and dietary treatment within each sampling day, pairwise PERMANOVA analysis implemented in QIIME2 was performed on an unweighted UniFrac distance matrix of 72 samples. The significance of PERMANOVA was obtained by 999 permutation tests.

Relative abundance of taxa for each sampling day and treatment were calculated at the phylum and genus levels.

The distinctive taxa between treatment groups were identified with linear discriminant analysis effect size (LEfSe) which was performed through Galaxy/Hutlab workflow platform (33). In order to predict the function of ileal mucosa microbiota, data analysis was performed through the Phylogenetic Investigation of Communities by Reconstruction of Unobserved States (PICRUSt) pipeline (34). Then, PICRUST output for the level 3 of the Kyoto Encyclopedia of Genes and Genomes (KEGG) were analyzed and illustrated with statistical analysis of the taxonomic and functional profiles (STAMP) software version 2.1.3 (35).

### Statistical Analyses

Statistical analysis for all data (except 16s sequencing) was performed using the ANOVA procedure of JMP software (2013) and significance between treatments (*P* < 0.05) determined by the LSD test. The statistical model for data analysis is outlined below:

Yij = μ + Ai + eij
Yij = measured value for each observation (data)
μ = grand mean
Ai = treatment effect
eij = experimental error.

## Results

### Mortality

Mortality was significantly (*P* = 0.011) reduced in PROB, PPEO and PC groups during the d 0–14 period compared to NC (Table 3). There were no significant differences in mortality between treatments during the overall (d 0–42) experimental period. However, compared to NC, PC and PPEO reduced d 0–42 mortality by 38 and 33%, respectively (although not statistically different likely due to variability among replicate pens).

**Table 3:** Effect of dietary probiotic (PROB) or probiotic/prebiotic/essential oil (PPEO) supplementation on mortality (%) of broiler chickens under a naturally occurring subclinical necrotic enteritis challenge.

**Treatments^1^**	**Time period (day)**					
	**0–8**	**9–14**	**0–14**	**15–28**	**29–42**	**0–42**
NC	2.91	2.40^a^	5.00^a^	1.00	2.13	7.50
PC	1.66	0.00^b^	1.66^b^	1.44	2.08	4.58
PROB	0.83	0.92^b^	1.66^b^	3.88	4.40	7.14
PPEO	0.83	0.00^b^	0.83^b^	0.96	4.10	5.00
*SEM*^2^	*0.76*	*0.46*	*0.87*	*1.00*	*1.46*	*1.65*
*P-value*	*0.201*	*0.002*	*0.011*	*0.146*	*0.696*	*0.450*

a,b *In each column, means with the same letter are not significantly different (P < 0.05)*.

1 *Treatments include: NC (negative control): corn-soybean meal basal diet; PC (positive control): NC + 20 g virginiamycin (453 g Stafac^®^20)/ton diet; PROB: NC + B. licheniformis spores at the level of 227 g/ton diet; PPEO: NC + 453 g/ton of a supplement containing B. licheniformis spores, mannan-oligosaccharides, β-glucans (1,3 and 1,6), capsaicin, and curcuma*.

2 *SEM, Standard error of means*.

### Necrotic Enteritis Lesion Scores

Typical with the challenge model applied, lesion scores were mostly prevalent in the duodenum portion and less pronounced in the distal sections of the small intestine (Table 4). Dietary supplementation of PROB and PPEO significantly (*P* = 0.013) reduced lesion scores in the duodenum compared to the NC birds. However, there was no difference in NE lesion scores for jejunum and ileum among the treatments.

**Table 4:** Effect of dietary probiotic (PROB) or probiotic/prebiotic/essential oil (PPEO) supplementation on intestinal lesion scores of broiler chickens under a naturally occurring subclinical necrotic enteritis challenge^1^.

**Treatments^2^**	**Small intestine section**		
	**Duodenum**	**Jejunum**	**Ileum**
NC	1.20^a^	0.58	0.00
PC	1.05^ab^	0.38	0.02
PROB	0.81^bc^	0.27	0.00
PPEO	0.64^c^	0.41	0.00
*SEM*^3^	*0.11*	*0.09*	*0.01*
*P-value*	*0.013*	*0.185*	*0.407*

a−c *In each column, means with the same letter are not significantly different (P <0.05)*.

1 *Data represent the mean value of 8 replicate pens of 3 birds/pen*.

2 *Treatments include: NC (negative control): corn-soybean meal basal diet; PC (positive control): NC + 20 g virginiamycin (453 g Stafac^®^20)/ton diet; PROB: NC + B. licheniformis spores at the level of 227 g/ton diet; PPEO: NC + 453 g/ton of a supplement containing B. licheniformis spores, mannan-oligosaccharides, β-glucans (1,3 and 1,6), capsaicin and curcuma*.

3 *SEM, Standard error of means*.

### Performance

All performance results are presented in Table 5. ADG was significantly higher (*P* < 0.001) for PPEO and PROB during the starter period (d 0–14) compared to NC and PC. In the grower period (d 15–28), PPEO and PC had significantly higher (*P* = 0.020) ADG compared to NC. Although ADG was statistically similar in all treatments during the finisher period (d 29–42), the PC and PPEO groups had significantly (*P* = 0.036) higher cumulative (d 0–42) ADG compared with NC. During the starter period, ADFI was significantly (*P* = 0.003) higher for PPEO and PROB compared to NC and PC; however, for the grower (d 15–28), finisher (d 29–42), and overall (d 0–42) experimental periods, feed intake was statistically similar (*P* > 0.05) among all the treatments. There was no difference in FCR among the treatments for the starter, grower or finisher periods. However, cumulative (d 0–42) FCR was significantly (*P* = 0.005) lower for all the treatments compared to NC.

**Table 5:** Effect of dietary probiotic (PROB) or probiotic/prebiotic/essential oil (PPEO) supplementation on broiler performance under a naturally occurring subclinical necrotic enteritis model^1^.

	**Dietary treatments^2^**				**Statistics**	
**Item^3^**	**NC**	**PC**	**PROB**	**PPEO**	***SEM*^4^**	***P-value***
d 0–8						
ADFI, g	26.87	26.46	27.53	27.42	*0.31*	*0.077*
ADG, g	21.46^b^	21.71^b^	22.87^a^	22.74^a^	*0.23*	*<0.001*
FCR, g/g	1.25^a^	1.22^ab^	1.20^b^	1.20^b^	*0.01*	*0.051*
d 9–14						
ADFI, g	61.63^c^	62.80^bc^	65.54^ab^	66.39^a^	*1.02*	*0.007*
ADG, g	41.26^c^	44.34^b^	46.52^a^	46.57^a^	*0.73*	*<0.001*
FCR	1.49	1.42	1.41	1.43	*0.02*	*0.119*
d 0–14						
ADFI, g	40.61^b^	41.00^b^	42.75^a^	43.18^a^	*0.52*	*0.003*
ADG, g	30.18^c^	31.52^b^	33.10^a^	33.06^a^	*0.34*	*<0.001*
FCR	1.34	1.30	1.29	1.30	*0.02*	*0.109*
d 15–28						
ADFI, g	121.99	125.80	123.23	125.38	*1.35*	*0.172*
ADG, g	77.35^b^	85.18^a^	80.35^ab^	83.68^a^	*1.79*	*0.020*
FCR	1.58	1.48	1.54	1.50	*0.03*	*0.121*
d 29–42						
ADFI, g	203.51	202.73	200.37	203.47	*2.44*	*0.783*
ADG, g	115.27	116.75	111.70	113.15	*2.99*	*0.650*
FCR	1.77	1.75	1.79	1.80	*0.04*	*0.797*
d 0–42						
ADFI, g	116.60	118.47	116.35	119.43	*1.33*	*0.338*
ADG, g	73.69^b^	77.61^a^	75.00^ab^	77.15^a^	*1.00*	*0.036*
FCR	1.58^a^	1.53^b^	1.55^b^	1.54^b^	*0.01*	*0.005*

a−c *In each column, means with the same letter are not significantly different (P < 0.05)*.

1 *Data represent the mean value of 8 replicate pens of 30 birds*.

2 *Treatments include: NC (negative control): corn-soybean meal basal diet; PC (positive control): NC + 20 g virginiamycin (453 g Stafac^®^20)/ton diet; PROB: NC + B. licheniformis spores at the level of 227 g/ton diet; PPEO: NC + 453 g/ton of a supplement containing B. licheniformis spores, mannan-oligosaccharides, β-glucans (1,3 and 1,6), capsaicin and curcuma*.

3 *ADFI, Average daily feed intake; ADG, average daily gain; FCR, feed conversion ratio*.

4 *SEM, Standard error of means*.

### mRNA Abundance of Tight Junction Proteins, Cytokines, and Master Regulators of Cell Metabolism

**Figure 1:**
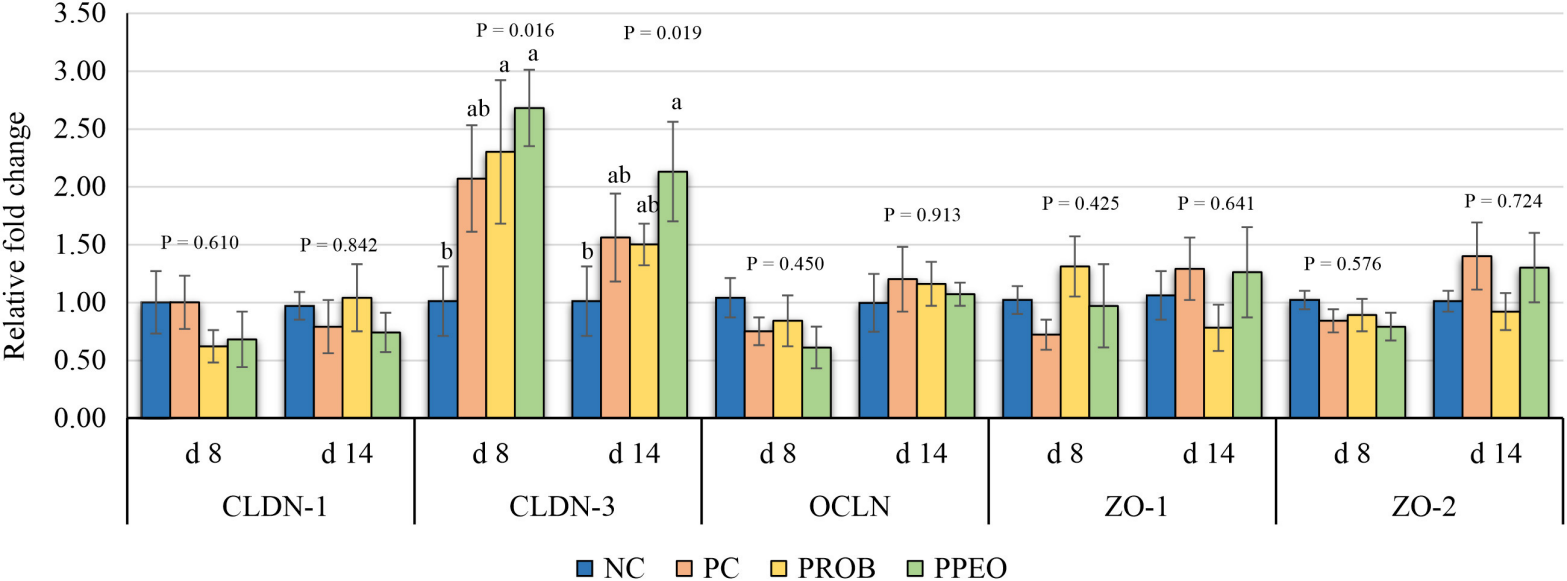
Relative mRNA abundance of tight junction proteins in the jejunum of broiler chickens on day (d) 8 and 14. Birds reared under a naturally occurring necrotic enteritis challenge model. Treatments include: NC (negative control): corn-soybean meal basal diet; PC (positive control): NC + 20 g virginiamycin (453 g Stafac^®^20)/ton diet; PROB (Probiotic): NC + *B. licheniformis* spores at the level of 227 g/ton diet; PPEO (probiotic/prebiotic/essential oil supplement): NC + 453 g/ton of a supplement containing *B. licheniformis* spores, mannan-oligosaccharides, β-glucans (1,3 and 1,6), capsaicin, and curcuma. Values are represented as a *n*-fold difference relative to the calibrator (NC). Results are given as means (*n* = 8) for each treatment. Error bars indicate standard errors. For each gene and sampling day, columns with different letters are significantly different (*P* < 0.05).

**Figure 2:**
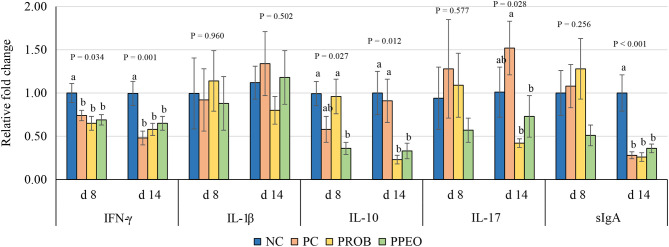
Relative mRNA abundance of interferon (IFN)-ɤ, interleukin (IL)-1β, IL-10, IL-17 and secretory immunoglobulin (sIg)A in the jejunum of broiler chickens on day (d) 8 and 14. Birds reared under a naturally occurring necrotic enteritis challenge model. Treatments include: NC (negative control): corn-soybean meal basal diet; PC (positive control): NC + 20 g virginiamycin (453 g Stafac^®^20)/ton diet; PROB (Probiotic): NC + *B. licheniformis* spores at the level of 227 g/ton diet; PPEO (probiotic/prebiotic/essential oil supplement): NC + 453 g/ton of a supplement containing *B. licheniformis* spores, mannan-oligosaccharides, β-glucans (1,3 and 1,6), capsaicin, and curcuma. Values are represented as a *n*-fold difference relative to the calibrator (NC). Results are given as means (*n* = 8) for each treatment. Error bars indicate standard errors. For each gene and sampling day, columns with different letters are significantly different (*P* < 0.05).

**Figure 3:**
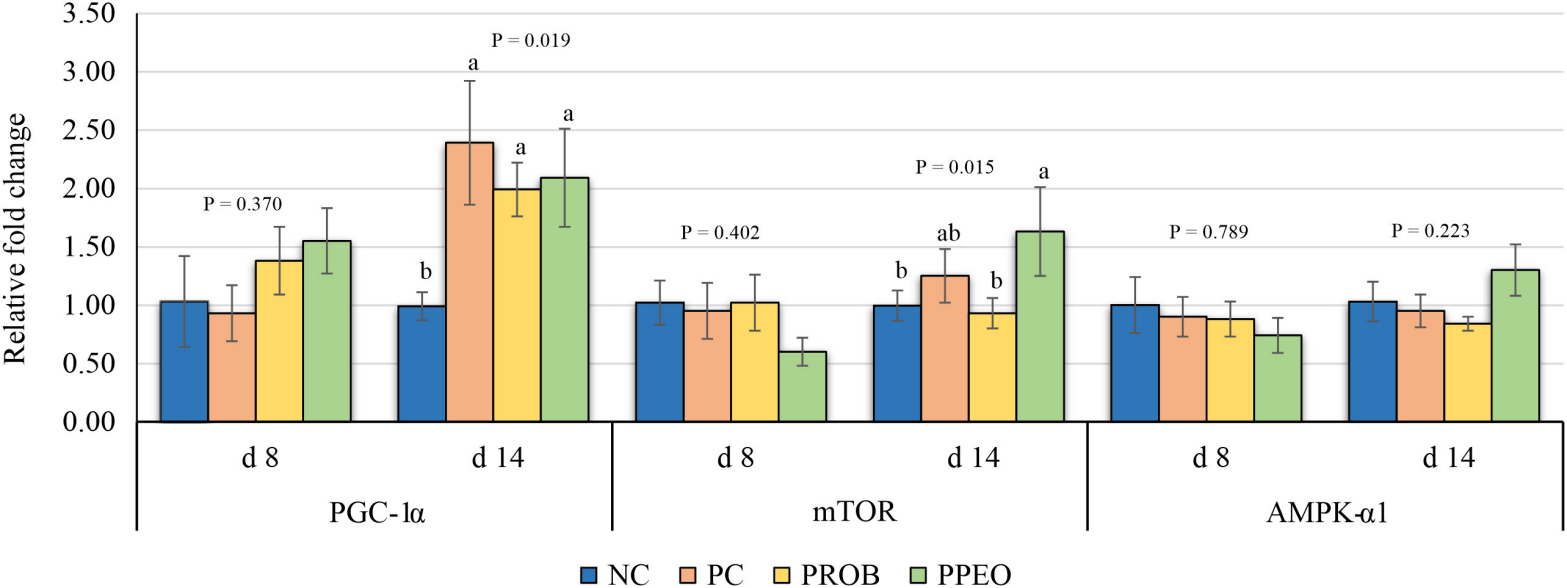
Relative mRNA abundance of peroxisome proliferator-activated receptor-gamma coactivator (PGC)-1α, mechanistic target of rapamycin (mTOR) and AMP activated protein kinase (AMPK)-α1 in the jejunum of broiler chickens on day (d) 8 and 14. Birds reared under a naturally occurring necrotic enteritis challenge model. Treatments include: NC (negative control): corn-soybean meal basal diet; PC (positive control): NC + 20 g virginiamycin (453 g Stafac^®^20)/ton diet; PROB (Probiotic): NC + *B. licheniformis* spores at the level of 227 g/ton diet; PPEO (probiotic/prebiotic/essential oil supplement): NC + 453 g/ton of a supplement containing *B. licheniformis* spores, mannan-oligosaccharides, β-glucans (1,3 and 1,6), capsaicin, and curcuma. Values are represented as a *n*-fold difference relative to the calibrator (NC). Results are given as means (*n* = 8) for each treatment. Error bars indicate standard errors. For each gene and sampling day, columns with different letters are significantly different (*P* < 0.05).

On d 8, mRNA abundance of CLDN-3 was significantly higher (*P* = 0.016) for the PROB and PPEO groups compared to NC, while on d 14 it was only higher (*P* = 0.019) in the PPEO group (Figure 1). However, abundance of CLDN-1, OCLN, ZO-1, and ZO-2 were not affected by any treatment. All treatment groups reduced (*P* = 0.034) mRNA abundance of IFN-γ compared to NC (Figure 2), while abundance of IL-1β was not affected by any treatment on d 8 and d 14. Abundance of sIgA was lower in PROB and PPEO groups compared to NC on d 14. Supplementation of PROB and PPEO reduced mRNA abundance of IL-17 compared to PC on d 14. Abundance of IL-10 was lower in PPEO compared to NC on d 8 (*P* = 0.027); but it was lower in PROB and PPEO on d 14 (*P* = 0.012). mRNA abundance of PGC-1α was higher (*P* = 0.019) for all the treatment groups compared to NC on d 14; while mTOR abundance was only higher (*P* = 0.015) for PPEO compared to NC on d 14 (Figure 3).

### Diversity and Composition of Microbiota

There was no difference between treatments with regard to alpha diversity on d 8; however, alpha diversity was different between NC and PPEO (*P* = 0.082) on d 42 (Supplementary Table 1; Supplementary Figures 1A, 2A). β-diversity PCoA plots showed a trend of separation of microbial communities among some groups on d 8 and d 42 (Supplementary Figures 1B, 2B).

PERMANOVA analyses on d 8 revealed that the composition of microbial communities in PPEO was different (*P* < 0.10) with NC and PC groups (Supplementary Table 2). On d 42, the composition of microbial communities in PC was different (*P* < 0.10) from PPEO and PROB groups (Supplementary Table 2).

The relative abundance of amplicon sequence variants of ileal scrapings microbiota was analyzed at different ranking levels from phylum to genus. The dominant phyla across the groups were *Firmicutes, Bacteroidetes, Proteobacteria, Actinobacteria, Tenericutes*, and *Epsilonbacteraeota* together contributing more than 99 and 82% of the whole phyla on d 8 and d 42, respectively (Table 6). Birds supplemented with additives had a higher abundance of *Firmicutes* and a lower abundance of *Bacteroidetes* compared to NC and PC on d 8. However, differences in relative abundance of the dominant bacterial phyla among the treatments decreased with age (Table 6).

**Table 6:** Comparison of microbial population in broiler chickens fed various dietary supplements on d 8 and 42 using 16S rRNA sequencing.

	**Dietary treatments^b^**			
**Relative abundance (%)**	**NC**	**PC**	**PROB**	**PPEO**
d 8				
*Actinobacteria*	1.95	2.72	0.93	0.54
*Bacteroidetes*	14.58	14.60	3.57	1.32
*Epsilonbacteraeota*	0.08	0.02	0.01	0.00
*Firmicutes*	81.82	76.35	94.21	97.67
*Proteobacteria*	1.39	5.99	0.95	0.39
*Tenericutes*	0.04	0.15	0.00	0.00
d 42				
*Actinobacteria*	0.23	0.46	0.14	0.16
*Bacteroidetes*	2.12	0.85	0.56	0.12
*Epsilonbacteraeota*	0.26	0.19	0.05	0.20
*Firmicutes*	78.61	75.33	85.92	80.83
*Proteobacteria*	1.94	5.23	0.74	4.29
*Tenericutes*	0.06	0.08	0.03	0.00

*Taxonomic diversity table shows the relative abundance (%) of taxa at the phylum level in each group. The relative abundance of each sample was calculated based on the amplicon sequence variants table rarefied to 10,891 and 29,818 reads per sample for d 8 and d 42, respectively^a^*.

a *Data represent the mean value of 1 bird/pen (8 birds/treatment)*.

b *Treatments include: negative control (NC): corn-soybean meal basal diet; positive control (PC): NC + 453 g virginiamycin (Stafac^®^20)/ton diet; probiotic (PROB): NC + 227 g B. licheniformis spores/ton diet; probiotic/prebiotic/essential oil supplement (PPEO): NC + 453 g of a supplement containing B. licheniformis spores, mannan-oligosaccharides, β-glucans (1,3 and 1,6), capsaicin and curcuma/ton diet*.

**Figure 4:**
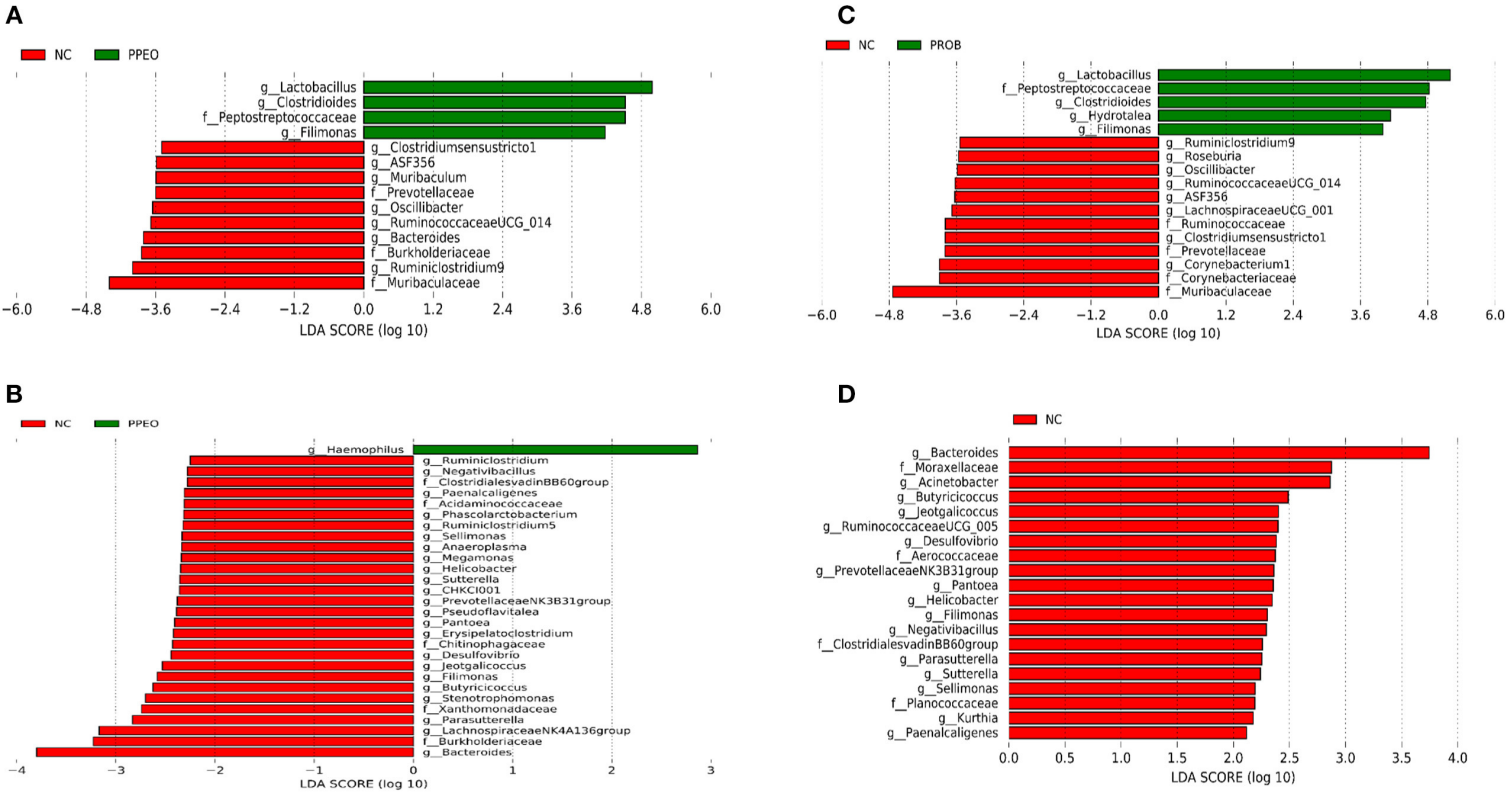
Taxonomic biomarkers highlighted by linear discriminant analysis effect size (LEfSE) (*P* ≤ 0.05 and LDA cutoff > 2.0) in ileal mucosa microbiota of broiler chickens challenged with a subclinical naturally occurring necrotic enteritis (*n* = 8/treatment). **(A,B)** Taxonomic biomarkers in NC vs. PPEO group on d 8 and d 42, respectively. **(C,D)** Taxonomic biomarkers in NC vs. PROB group on d 8 and d 42, respectively. NC (negative control): corn-soybean meal basal diet; PPEO (probiotic/prebiotic/essential oil supplement): NC + 453 g/ton of a supplement containing *B. licheniformis* spores, mannan-oligosaccharides, β-glucans (1,3 and 1,6), capsaicin, and curcuma; PROB (Probiotic): NC + 227 g *B. licheniformis* spores/ton diet.

**Figure 5:**
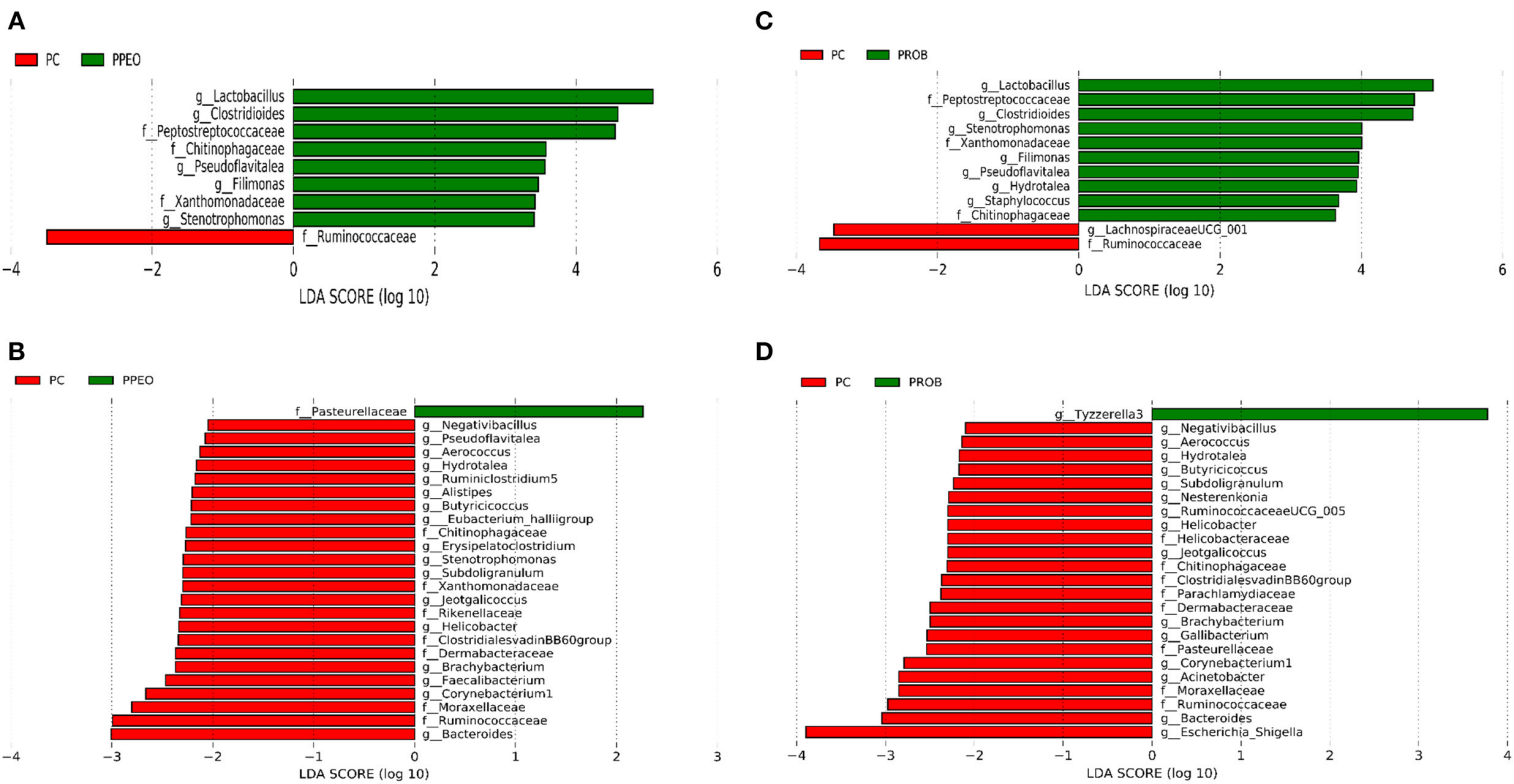
Taxonomic biomarkers highlighted by linear discriminant analysis effect size (LEfSE) (*P* ≤ 0.05 and LDA cutoff > 2.0) in ileal mucosa microbiota of broiler chickens challenged with a subclinical naturally occurring necrotic enteritis (*n* = 8/treatment). **(A,B)** Taxonomic biomarkers in PC vs. PPEO group on d 8 and d 42, respectively. **(C,D)** Taxonomic biomarkers in PC vs. PROB group on d 8 and d 42, respectively. PC (positive control): basal diet (corn-soybean meal) + 20 g virginiamycin (453 g Stafac^®^20)/ton diet; PPEO (probiotic/prebiotic/essential oil supplement): basal diet + 453 g/ton of a supplement containing *B. licheniformis* spores, mannan-oligosaccharides, β-glucans (1,3 and 1,6), capsaicin, and curcuma. PROB (Probiotic): basal diet + 227 g *B. licheniformis* spores/ton diet.

On d 8, relative abundance of *Rominiclostridium 9, Oscillibacter, RuminococcaceaeUCG_014, ASF356, Clostridium sensu stricto 1, Prevotellacea*, and *Muribaculaceae* was higher in NC compared to PROB and PPEO groups (Figures 4A,C). On the other hand, relative abundance of *Lactobacillus* was lower in the NC birds compared to the PROB and PPEO treatments. On d 42, relative abundance of *Bacteroides, Clostridiales vadin BB60 group, Prevotellaceae* NK3B31 group, *Desulfovibrio, Butyricicoccus, Helicobacter, Sellimonas*, and *Filimonas* was higher in NC compared to PROB and PPEO groups (Figures 4B,D). Interestingly, relative abundance of *Lactobacillus* was significantly lower (Figures 5A,C) while relative abundance of *Escherichia*-*Shigella* was numerically higher (data not shown) in PC compared to PPEO and PROB groups on d 8. Furthermore, relative abundance of several bacterial genera and families were different in PC compared to PROB and PPEO groups on d 42 (Figures 5B,D).

### Predicted Functions of Ileal Mucosal Microbiota

**Figure 6:**
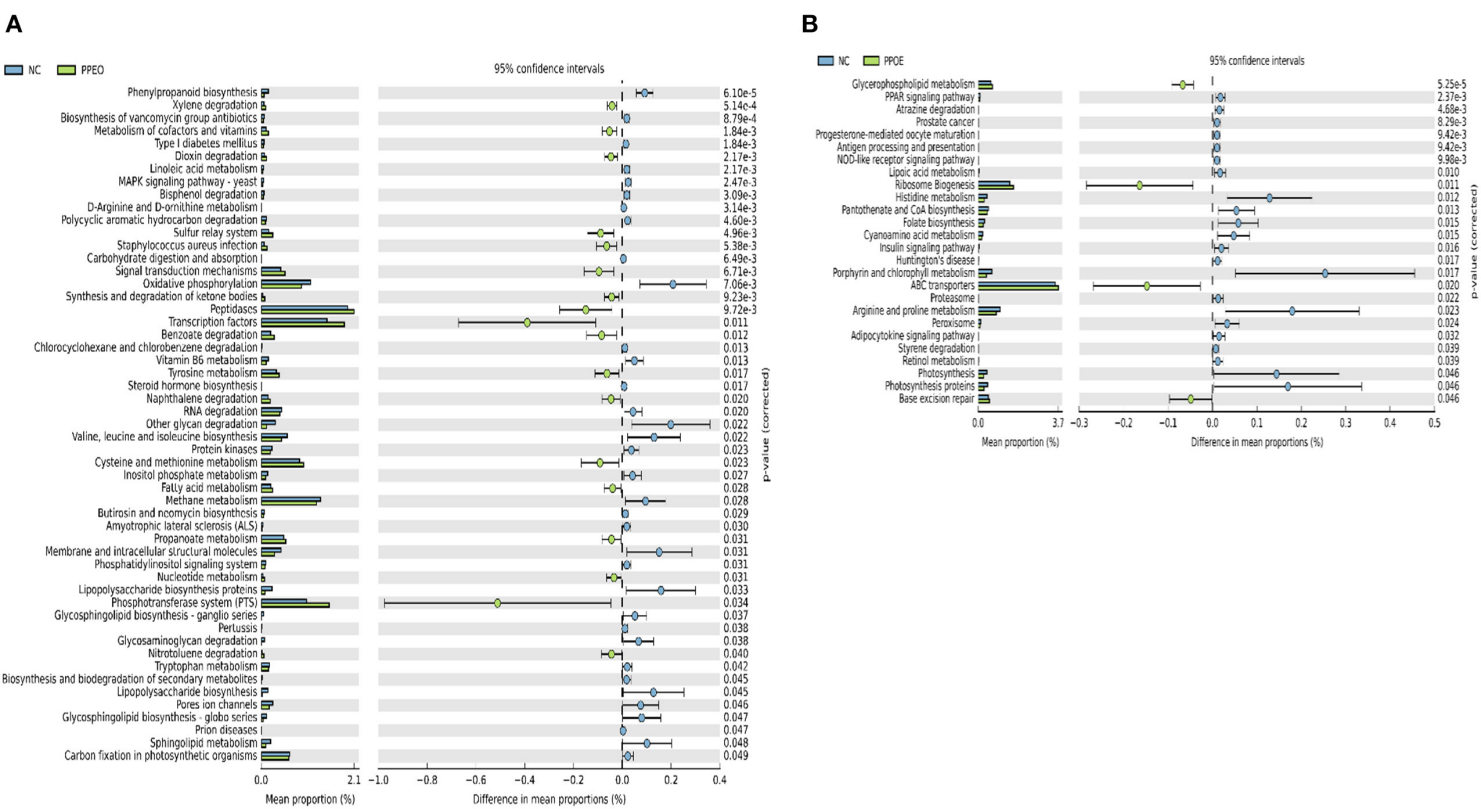
Predicted functions of ileal mucosa microbiota in broiler chickens challenged with a subclinical naturally occurring necrotic enteritis at KEGG levels 3 (*n* = 8/treatment). **(A,B)** Differentially regulated metabolic pathways in NC vs. PPEO group on d 8 and d 42, respectively. NC (negative control): corn-soybean meal basal diet; PPEO (probiotic/prebiotic/essential oil supplement): NC + 453 g/ton of a supplement containing *B. licheniformis* spores, mannan-oligosaccharides, β-glucans (1,3 and 1,6), capsaicin, and curcuma.

**Figure 7:**
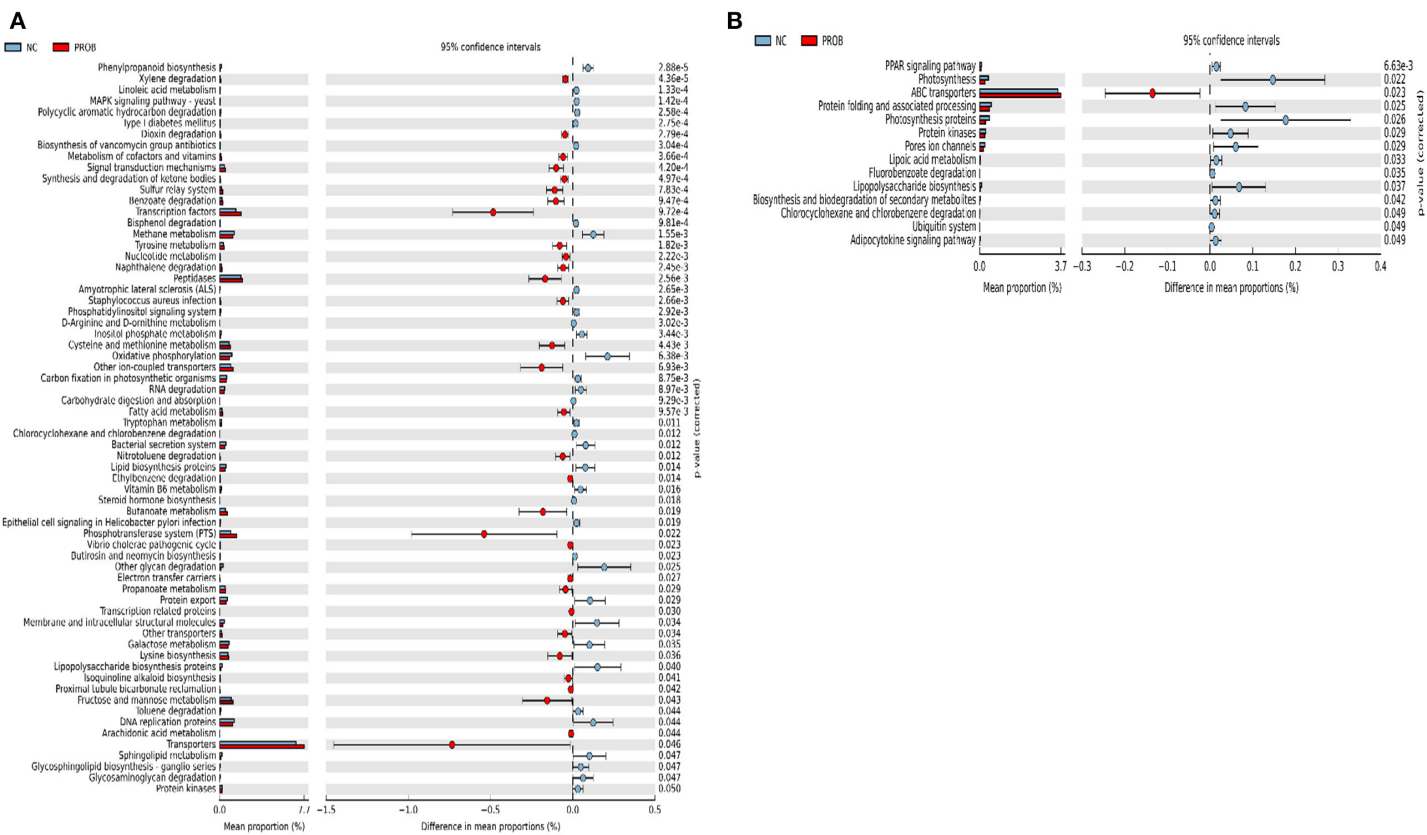
Predicted functions of ileal mucosa microbiota in broiler chickens challenged with a subclinical naturally occurring necrotic enteritis at KEGG levels 3 (*n* = 8/treatment). **(A,B)** Differentially regulated metabolic pathways in NC vs. PROB group on d 8 and d 42, respectively. NC (negative control): corn-soybean meal basal diet; PROB (Probiotic): NC + 227 g *B. licheniformis* spores/ton diet.

Prediction of microbial functions showed distinctive results among the treatment groups. There were 53 and 26 pathways at KEGG level 3 with distinctive enrichment between NC and PPEO groups on d 8 and d 42, respectively (Figures 6A,B). There were 66 and 14 pathways at KEGG level 3 with distinctive enrichment between NC and PROB groups on d 8 and d 42, respectively (Figures 7A,B). Among these, the NC microbiota had higher numbers of functional genes involved in lipopolysaccharide (LPS) biosynthesis, LPS biosynthesis proteins, DNA replication proteins, membrane and intracellular structural molecules, protein kinases, oxidative phosphorylation, and bacterial secretion system compared to PROB and PPEO. Interestingly, functional genes involved in both butanoate metabolism and propanoate metabolism were enriched in PROB compared to NC, while PPEO only enriched functional genes involved in propanoate metabolism compared to NC on d 8.

**Figure 8:**
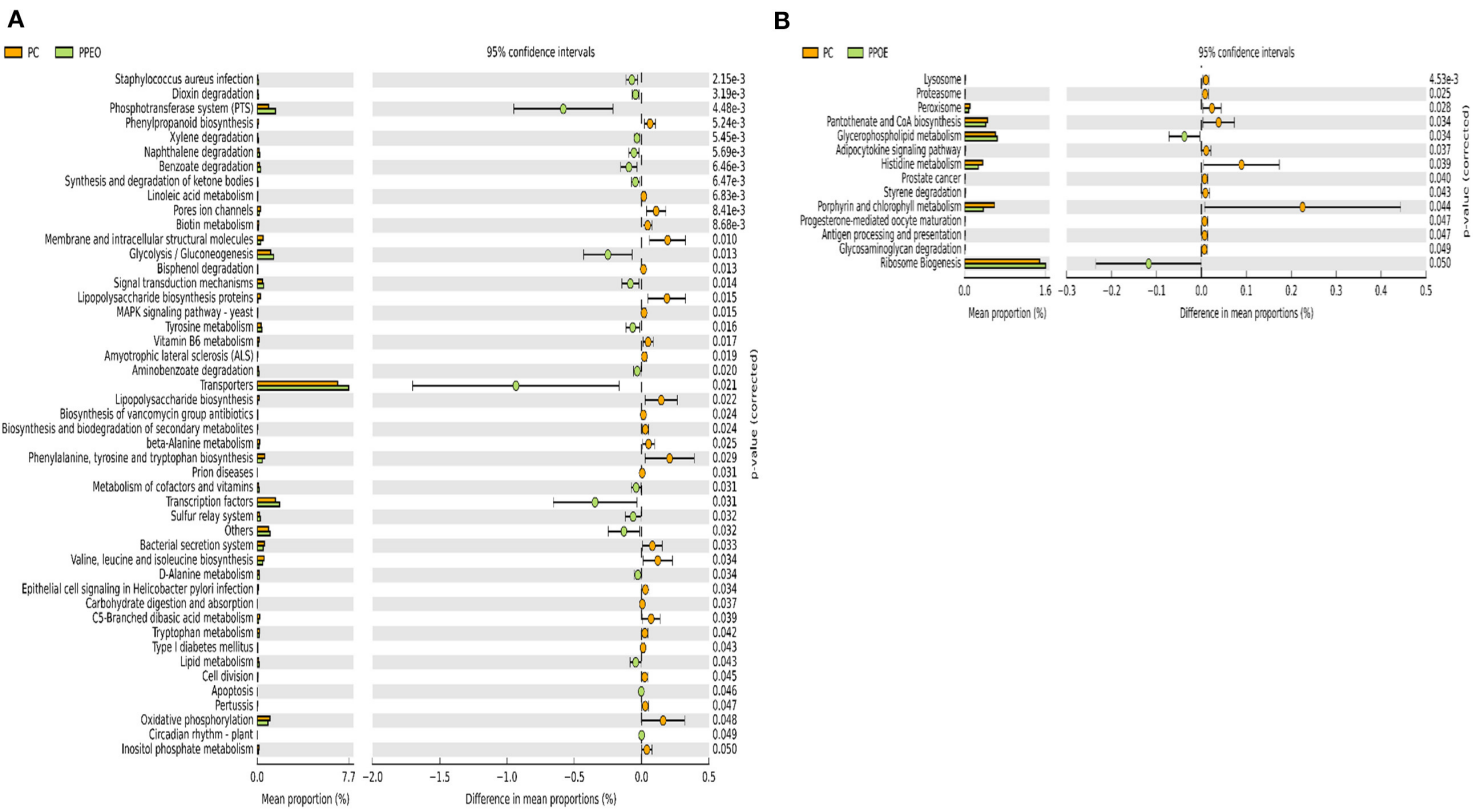
Predicted functions of ileal mucosa microbiota in broiler chickens challenged with a subclinical naturally occurring necrotic enteritis at KEGG levels 3 (*n* = 8/treatment). **(A,B)** Differentially regulated metabolic pathways in PC vs. PPEO group on d 8 and d 42, respectively. PC (positive control): basal diet (corn-soybean meal) + 20 g virginiamycin (453 g Stafac^®^20)/ton diet; PPEO (probiotic/prebiotic/essential oil supplement): basal diet + 453 g/ton of a supplement containing *B. licheniformis* spores, mannan-oligosaccharides, β-glucans (1,3 and 1,6), capsaicin, and curcuma.

**Figure 9:**
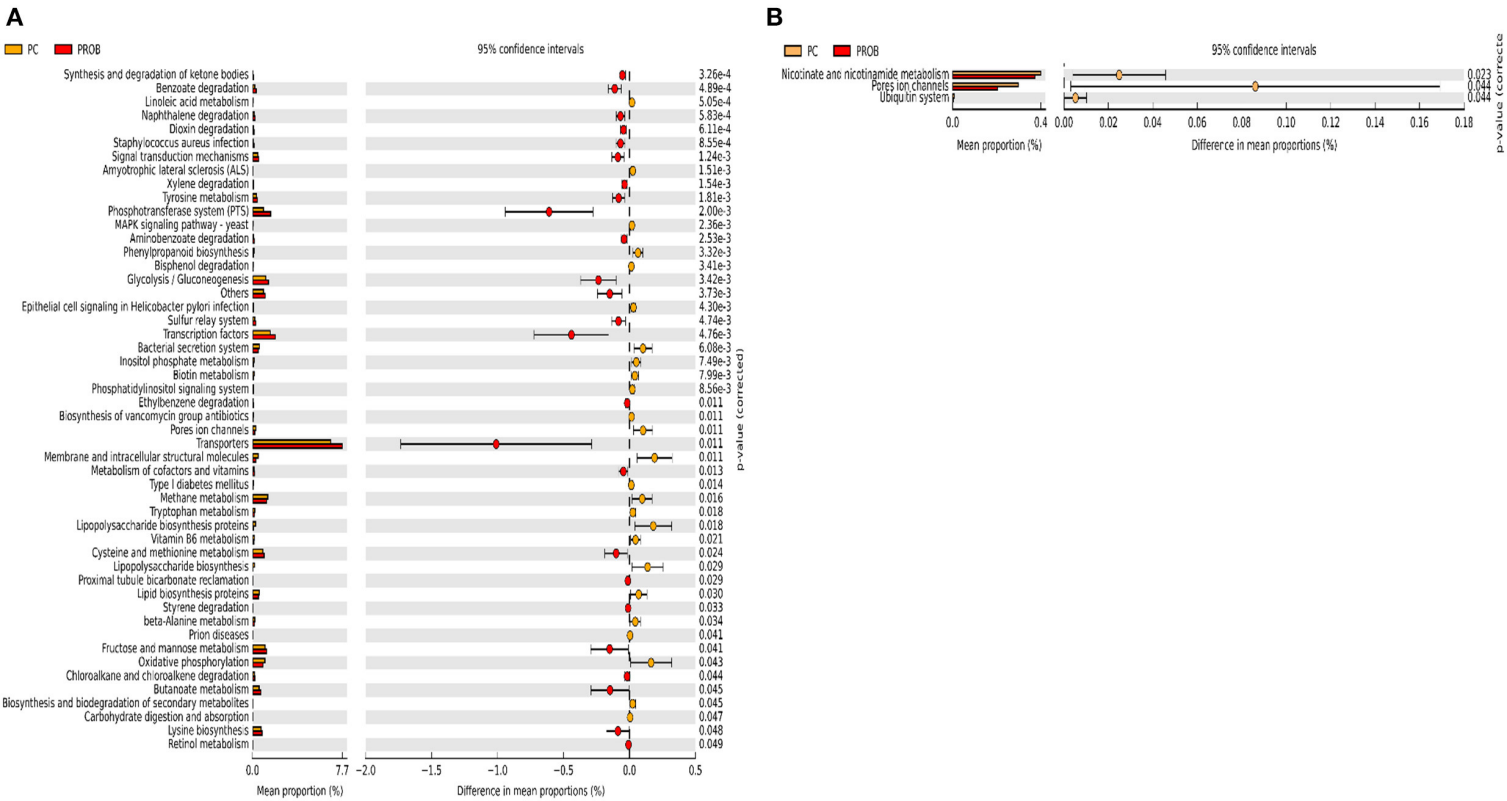
Predicted functions of ileal mucosa microbiota in broiler chickens challenged with a subclinical naturally occurring necrotic enteritis at KEGG levels 3 (*n* = 8/treatment). **(A,B)** Differentially regulated metabolic pathways in PC vs. PROB group on d 8 and d 42, respectively. PC (positive control): basal diet (corn-soybean meal) + 20 g virginiamycin (453 g Stafac^®^20)/ton diet; PROB (Probiotic): basal diet + 227 g *B. licheniformis* spores/ton diet.

There were 47 and 14 pathways at KEGG level 3 with distinctive enrichment between the PC and PPEO groups on d 8 and d 42, respectively (Figures 8A,B), while 50 and 3 pathways with distinctive enrichment were identified between the PC and PROB groups on d 8 and d 42, respectively (Figures 9A,B). Among these, the PROB group had higher numbers of functional genes involved in lysine biosynthesis, butanoate metabolism, retinol metabolism, glycolysis/gluconeogenesis and synthesis and degradation of ketone bodies; while LPS biosynthesis, LPS biosynthesis proteins, bacterial secretion system, lipid biosynthesis proteins, and carbohydrate digestion and absorption were enriched in PC compared to PPEO and PROB groups on d 8. On d 42, lysosome, proteasome, peroxisome, antigen processing and presentation were enriched in PC compared to PPEO; however, ribosome biogenesis and glycerophospholipid metabolism were enriched in PPEO.

## Discussion

Necrotic enteritis occurs in clinical (acute) or subclinical (chronic) form (2, 3). During subclinical NE, chronic damage to the intestinal mucosa leads to poor performance (reduced FI, weight gain and eventually increased FCR) with or without low mortality (4, 5). The NE challenge in this study was subclinical as manifested by low mortality and lesion scores in all the treatments. Better performance in PROB and PPEO groups may be due to the modulated immune responses (lower mRNA abundance of IFN-γ, sIgA, IL-10, and IL-17), better gut health (lower lesion scores and higher mRNA abundance of CLDN-3), and modified energy and protein metabolism (higher abundance of PGC-1α and mTOR). Immune responses are energy demanding processes that divert nutrients from growth, subsequently reducing performance. Therefore, in PROB and PPEO birds, nutrients could have been directed toward growth rather than mounted immune responses in NC birds. Previously, *B. licheniformis* spores reduced NE mortality and lesion score, and improved FCR compared to the *C. perfringens*-challenged non-medicated birds (36). Furthermore, supplementation of *B. subtilis* to the diet of broiler chickens challenged with NE significantly decreased lesion scores and improved FCR compared to challenged control birds (37–39).

Gut health plays a pivotal role in the chickens' ability to reach their genetic potential and low FCR, while diminished feed efficiency and profitability would be the result of enteric diseases such as NE (4, 5). In this study, birds in the PPEO and PROB groups had significantly lower NE lesion scores in the duodenum compared to NC. Lower lesion scores reflect a stronger gut barrier against *C*. *perfringens* and may explain the lower mortality rates in those birds compared to NC birds during the early (d 0–14) challenge period (5% compared to 1.66 and 0.8%, respectively). An intact intestinal epithelium prevents entry of potential pathogens and leads to optimal health and performance of birds as a result of proper nutrient absorption and utilization (40). Tight junction proteins are critical components of gut integrity and make up a barrier in the paracellular space. However, these barriers have a dynamic structure that remodel in response to signals from the cells or external stimuli such as nutrients, and pathogenic and commensal bacteria (13). Furthermore, TJ proteins are binding sites for *C*. *perfringens* enterotoxins (CPE) (41), and understanding their changes during NE is a promising aspect of research. In this study, supplementation of PPEO led to higher mRNA abundance of CLDN-3 on d 8 and d 14 compared to NC, while CLDN-3 abundance was higher in PROB fed birds only on d 8. Particularly, the CLDN family proteins are structural components of TJ proteins and characterized by their extracellular domain to which CPE attach and influence gut integrity (15, 41). Higher abundance of CLDN-3 in PROB and PPEO groups coincided with lower lesion scores and better FCR in these groups. Therefore, one explanation would be that these products alleviated the negative effects of NE, thus the higher mRNA abundance of CLDN-3. In other words, lower abundance of CLDN-3 in the NC birds might be a defense mechanism to reduce the attachment sites for CPE in order to reduce its negative effects. We have previously shown the possibility of TJ modification in chickens through supplementation of probiotics under a field-like NE challenge (42). Curcumin (a component of PPEO) can increase the mRNA abundance of ZO-1 and CLDN-1 in Caco-2 cells and alleviate IL-1β-induced disorganization of ZO-1, CLDN-1, and CLDN-7 in intestinal epithelial cells, leading to improved barrier function and reduced paracellular permeability (43, 44). This might be a reason for the effect of PPEO on CLDN-3 mRNA abundance on d 14, while there was no effect of PROB alone.

Higher lesion scores in the NC birds was accompanied by higher relative abundance of *Rominiclostridium 9, Oscillibacter, RuminococcaceaeUCG_014, ASF356, Clostridium sensu stricto 1, Prevotellacea*, and *Muribaculaceae* compared to PROB and PPEO on d 8. Conversely, relative abundance of *Lactobacillus* was lower in NC compared to other treatments except PC. *Prevotellaceae* can degrade mucus oligosaccharides resulting in the disruption of intestinal mucosal barrier and intestinal inflammation (45). In addition, higher proportion of *Prevotellaceae* has been detected in the microbiota of NLRP6 KO mice with high risk for colitis (46). Higher relative abundance of *Prevotellaceae* in the NC group might have led to the disruption of the mucosal barrier rendering it prone to bacterial pathogens, which coincided with higher FCR and lesion scores in this group. Several studies reported the suppression of *Lactobacillus* at the time of *C*. *perfringens* challenge (6, 24, 47). Certain pathogenic bacteria have the ability to attach to the intestinal mucosa; however, probiotic bacteria such as *Lactobacillus* and *Bifidobacterium* prevent adherence of pathogens to the intestinal mucosa via competitive exclusion thus displacing pathogenic bacteria (48, 49). Accordingly, higher relative abundance of *Lactobacillus* might have led to lower NE lesions in the jejunum of broilers in the PROB and PPEO groups. A mixed *C*. *perfringens* and *Eimeria* challenge led to a significant increase in *Clostridium sensu stricto 1* and reduction in *Lactobacillus* with the concurrent increase in NE lesions (50); the authors concluded that overgrowth of *C*. *sensu stricto 1* is associated with NE. As reported earlier, supplementation of feed additives in this study reduced relative abundance of *C*. *sensu stricto 1* in the ileal mucosal scrapings of broiler chickens, which might be a reason for better gut health and lower NE lesions in these groups. *C*. *perfringens* infection decreased (numerically) the relative abundance of the *Peptostreptococcaceae* family, and increased that of the *Enterobacteriaceae* family, mainly composed of *Escherichia-Shigella*, in the ileum of broiler chickens (24). This group of bacteria is mainly composed of opportunistic pathogens and higher presence might be an indicator of disrupted gut health that allows the overgrowth of such pathogens. Further, *C*. *perfringens* and its co-infection with *Eimeria* increased relative abundance of *Escherichia-Shigella* in the jejunum of broiler chickens (50). In addition, other reports indicated a positive correlation between the relative abundance of *Escherichia*-*Shigella* with NE occurrence in broiler chickens (51). In this study, the relative abundance of *Lactobacillus* was significantly lower, while relative abundance of *Escherichia-Shigella* was numerically higher in the PC birds compared to other groups. Supplementation of virginiamycin in combination with monensin to broiler chickens' diet increased *E*. *coli, Coprococcus*, and *Anaeroflum*, while *Roseburia, Lactobacillus*, and *Enterococcus* decreased in the ceca (52). Previous study showed that a probiotic (*L. acidophilus*) improved intestinal health by numerically decreasing the relative abundance of Escherichia-Shigella in the ileum (24).

*Lactobacillus* are among the predominant bacterial genera in the gastrointestinal tract of broiler chickens (53), and provide both direct and indirect beneficial effects. The direct effects include immunomodulation via attachment and interaction with enterocytes, antagonistic activity against pathogens by production of lactate thus lowering pH and making the gastrointestinal tract environment unsuitable for acid-sensitive pathogens, and competitive exclusion mechanisms along with the production of bacteriostatic and bactericidal substances (54–56). Supplementation of PROB and PPEO increased the relative abundance of *Lactobacillus* in the ileal mucosa, thereby possibly contributing to their positive effect on gut health and performance of the birds. Likewise, dietary supplementation of a probiotic to broiler chickens challenged with NE increased abundance of *Lactobacilliaceae* and *Clostridiaceae* families in the cecal digesta compared with the challenged control group (39).

Supplementation of PROB enriched the predicted metabolism of butanoate and propanoate in the ileal bacteria compared to NC on d 8. This might be due to increased relative abundance of *Firmicutes* in PROB compared to NC. Butyrate producers mostly belong to the *Firmicutes* phylum (57). In addition, *Bifidobacterium* and *Lactobacillus* are the substrate providers for butyrate producers (58). Optimal butyrate production relies on the presence of butyrate-producing bacteria and various others including lactate-producing bacteria that cross-feed butyrate producers (59, 60). Butyrate could enhance epithelial regeneration by stimulating villus growth; however, it does not inhibit *C*. *perfringens* (61, 62). Absorption of butyrate and propionate by chicken cecal mucosa could improve host energy metabolism and improve performance (63). Similar to this study, supplementation of *B*. *licheniformis* enriched butanoate metabolism in the microbiota of broiler chickens challenged with NE compared to challenged non-supplemented group (64). Further, dietary supplementation of a prebiotic (but not probiotic and symbiotic) to laying hens enriched cecal microbial genes involved in butanoate and propanoate metabolism (63).

The essential oils thyme and anise decreased *C*. *perfringens* and *E*. *coli* counts in the small intestines, along with decreased intestinal lesion scores and significantly better FCR (65). Essential oils are constituents of the PPEO supplement used in this study and we observed lower relative abundance of *Escherichia*-*Shigella* in the ileum of birds in this treatment group. The probiotic *B*. *subtilis* DSM 32315 increased the abundance of *Firmicutes* while reduced the abundance of *Bacteroidetes* in the ceca of broiler chickens (66). Furthermore, this probiotic decreased the abundance of potentially harmful bacteria such as *Vampirovibrio, Escherichia*-*Shigella*, and *Parabacteroides* (66). Likewise, supplementation of PROB to the diet led to increased *Firmicutes* abundance and reduced *Bacteroidetes* abundance in the ileal mucosa of broiler chickens.

Mannooligosaccharides constitute one component of PPEO. *In vitro* studies showed high levels of propionate production from mannooligosaccharides fermentation (67), and production of short chain fatty acids reduced cecum pH, thus inhibiting the growth of *Enterobacteriaceae* (which includes *Escherichia-Shigella*) (68). Lower relative abundance of *Escherichia-shigella* in the PPEO and PROB groups compared to PC was associated with the reduction of LPS biosynthesis, LPS biosynthesis proteins, and bacterial secretion system in PROB and PPEO groups compared to NC and PC as indicated by KEGG pathway. LPS induce mucosal immune responses (69); thus, such responses to the overgrowth of opportunistic bacteria due to the dysbiosis caused by NE challenge might have been attenuated by PROB and PPEO supplementation.

Supplementation of PPEO but not PROB significantly increased mRNA abundance of PGC-1α and mTOR on d 14 compared to NC. Mitochondrial function is regulated by PGC-1α. PGC-1α modulates oxidative metabolism by increasing mitochondrial function and at the same time minimizing the build-up of oxidative respiration by-products such as reactive oxygen species (70). mTOR regulates protein and lipid synthesis, thus its signaling is of significant/central importance in regulating cell metabolism, growth, proliferation and survival (71). Renewal of intestinal epithelial cells along the crypt-villus axis is regulated by the mTOR signaling pathway. This may partly be due to its ability to affect the antioxidant capacity and protein synthesis of intestinal epithelial cells (9, 10). Metabolism is controlled by sensing the internal (host) and external (presence of bacteria and nutrients in the gut lumen) signals that are integrated by mTOR and PGC-1α (71). These signals might lead to activation and proliferation of immune cells (such as T cells) leading to retarded growth as a consequence of a strong immune response (72). Therefore, mounted immune responses in the NC birds might be the cause of reduced ADG and increased FCR in this group. During injury or infection, archetypal pro-inflammatory cytokines (IL-1 and TNF-α) are rapidly released. Pro-inflammatory cytokines, including IL-1β and IFN-γ, could disrupt intestinal barrier function by affecting TJ structure (20, 43, 73). Here, IFN-y mRNA abundance was lower in PPEO and PROB compared to the NC group, demonstrating the protective effect of these supplements in reducing the potential damage caused by pro-inflammatory cytokines to the intestinal barrier.

## Conclusion

Based on the presented findings, we can conclude that under a naturally occurring subclinical NE challenge model, supplementation of a PROB or PPEO to broiler diets could reduce d 8 lesion scores and decrease FCR during the cumulative (d 0–42) grow-out period. Additionally, birds fed diets supplemented with PPEO had significantly higher ADG during d 0–42. These changes might be due to higher mRNA abundance of TJ proteins, PGC-1α and mTOR, and modulated cytokine responses in these groups. Ileal mucosa microbial profiling and functional analysis revealed taxonomic and functional signatures associated with dietary supplementation of PPEO or PROB compared to NC and PC birds. Overall, compared to probiotic alone, concurrent supplementation of probiotic/prebiotic/essential oils resulted in better cumulative performance under this naturally occurring NE challenge model.

## Data Availability Statement

The datasets generated for this study can be found in the European Nucleotide Archive, Accession No. PRJEB40959.

## Ethics Statement

The animal study was reviewed and approved by the Virginia Tech Institutional Animal Care and Use Committee.

## Author Contributions

NE, AC, and MW conducted the study. NE contributed to research design, performed laboratory, microbiota and statistical analyses, and drafted the manuscript. EK contributed to research design. RD was the principal investigator involved in every aspect of this study. All authors contributed to the article and approved the submitted version.

## Conflict of Interest

EK was employed by Huvepharma Inc., but no longer works with the company. The remaining authors declare that the research was conducted in the absence of any commercial or financial relationships that could be construed as a potential conflict of interest.
