# Supplementary material for: Effect of Probiotics and Multi-Component Feed Additives on Microbiota, Gut Barrier and Immune Responses in Broiler Chickens During Subclinical Necrotic Enteritis
Source: Front Vet Sci. 2020 Nov 26;7:572142. doi: 10.3389/fvets.2020.572142 (PMC7725796; doi:10.3389/fvets.2020.572142)
Supplement: Supplementary file 1 [file Data_Sheet_1.docx]

**TABLE S1**. Pairwise Kruskal-Wallis test for phylogenetic diversity of treatment groups using Shannon index analysis on d 8 and 42.^1^

|  | **Group 1** | **Group 2** | **H** | ***P-value*** | **q-value** |
| --- | --- | --- | --- | --- | --- |
| d 8 | NC | PC | 0.099 | 0.752 | 0.752 |
|  |  | PPEO | 1.334 | 0.247 | 0.743 |
|  |  | PROB | 0.275 | 0.599 | 0.752 |
|  | PC | PPEO | 0.099 | 0.752 | 0.752 |
|  |  | PROB | 0.397 | 0.528 | 0.752 |
|  | PPEO | PROB | 2.161 | 0.141 | 0.743 |
| d 42 | NC | PC | 0.656 | 0.417 | 0.501 |
|  |  | PPEO | 3.013 | 0.082 | 0.230 |
|  |  | PROB | 2.6250 | 0.105 | 0.230 |
|  | PC | PPEO | 2.481 | 0.115 | 0.230 |
|  |  | PROB | 0.705 | 0.400 | 0.501 |
|  | PPEO | PROB | 0.397 | 0.528 | 0.528 |

^1^ Treatments include: negative control (NC): corn-soybean meal basal diet; positive control (PC): NC + 20 g virginiamycin (453 g Stafac^®^20)/ton diet; probiotic (PROB): NC + 227 g *B. licheniformis* spores/ton diet; probiotic/prebiotic/essential oil supplement (PPEO): NC + 453 g of a supplement containing B. licheniformis spores, mannan-oligosaccharides, β-glucans (1,3 and 1,6), capsaicin and curcuma/ton diet.

^2^ *P*-values were calculated based on 999 permutation tests.


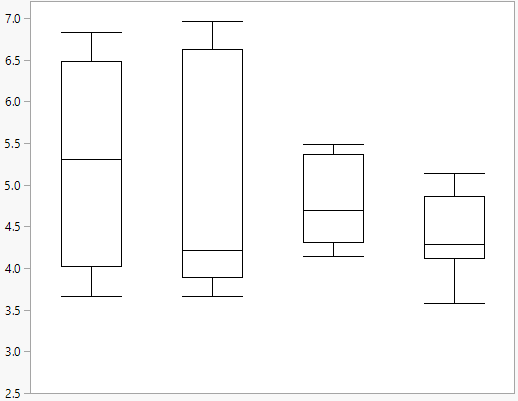


**(A)**

Shannon index

NC

PC

PROB

PPEO

**(B)**


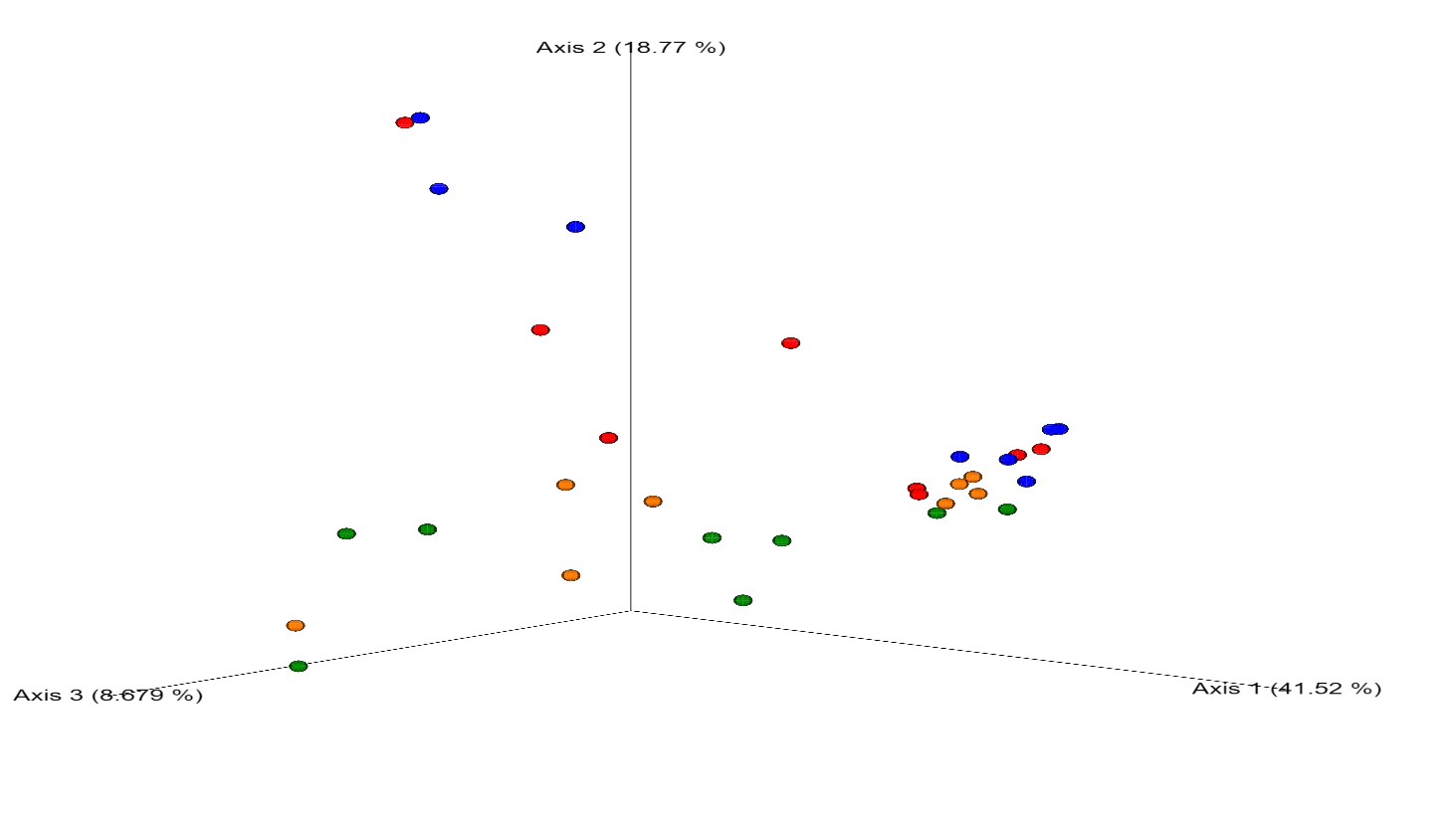


NC

PC

PPEO

PROB

**FIGURE S1**. Comparison of microbial population in broiler chickens fed various dietary supplements on d 8. (**A**) Shannon’s diversity index of each treatment group. Boxplots show the quartiles, median, and extremities of the values. (**B**) 3D PCoA plots based on weighted UniFrac distance matrix (bray-curtis). Each sphere represents a sample. Treatments include: negative control (NC): corn-soybean meal basal diet; positive control (PC): NC + 20 g virginiamycin (453 g Stafac^®^20)/ton diet; probiotic (PROB): NC + 227 g *B. licheniformis* spores/ton diet; probiotic/prebiotic/essential oil supplement (PPEO): NC + 453 g of a supplement containing *B. licheniformis* spores, mannan-oligosaccharides, β-glucans (1,3 and 1,6), capsaicin and curcuma/ton diet.

**TABLE S2**. Pairwise PERMANOVA statistic based on unweighted UniFrac distance matrix on d 8 and 42.^1^

|  | **Group 1** | **Group 2** | **pseudo-F** | ***P*-value**^2^ | **q-value** |
| --- | --- | --- | --- | --- | --- |
| d 8 | NC | PC | 0.549 | 0.887 | 0.887 |
|  |  | PPEO | 1.922 | 0.056 | 0.246 |
|  |  | PROB | 1.270 | 0.243 | 0.291 |
|  | PC | PPEO | 1.745 | 0.082 | 0.246 |
|  |  | PROB | 1.476 | 0.140 | 0.280 |
|  | PPEO | PROB | 1.205 | 0.212 | 0.291 |
| d 42 | NC | PC | 0.798 | 0.636 | 0.636 |
|  |  | PPEO | 1.053 | 0.374 | 0.561 |
|  |  | PROB | 1.374 | 0.115 | 0.230 |
|  | PC | PPEO | 1.742 | 0.070 | 0.230 |
|  |  | PROB | 1.674 | 0.096 | 0.230 |
|  | PPEO | PROB | 0.955 | 0.515 | 0.618 |

^1^ Treatments include: negative control (NC): corn-soybean meal basal diet; positive control (PC): NC + 20 g virginiamycin (453 g Stafac^®^20)/ton diet; probiotic (PROB): NC + 227 g *B. licheniformis* spores/ton diet; probiotic/prebiotic/essential oil supplement (PPEO): NC + 453 g of a supplement containing B. licheniformis spores, mannan-oligosaccharides, β-glucans (1,3 and 1,6), capsaicin and curcuma/ton diet.

^2^ *P*-values were calculated based on 999 permutation tests.

**
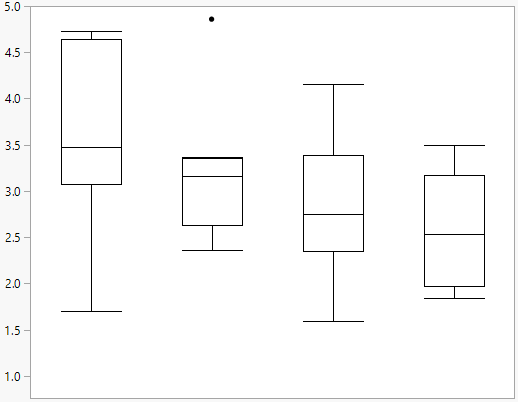
**

**(A)**

Shannon index

NC

PC

PROB

PPEO


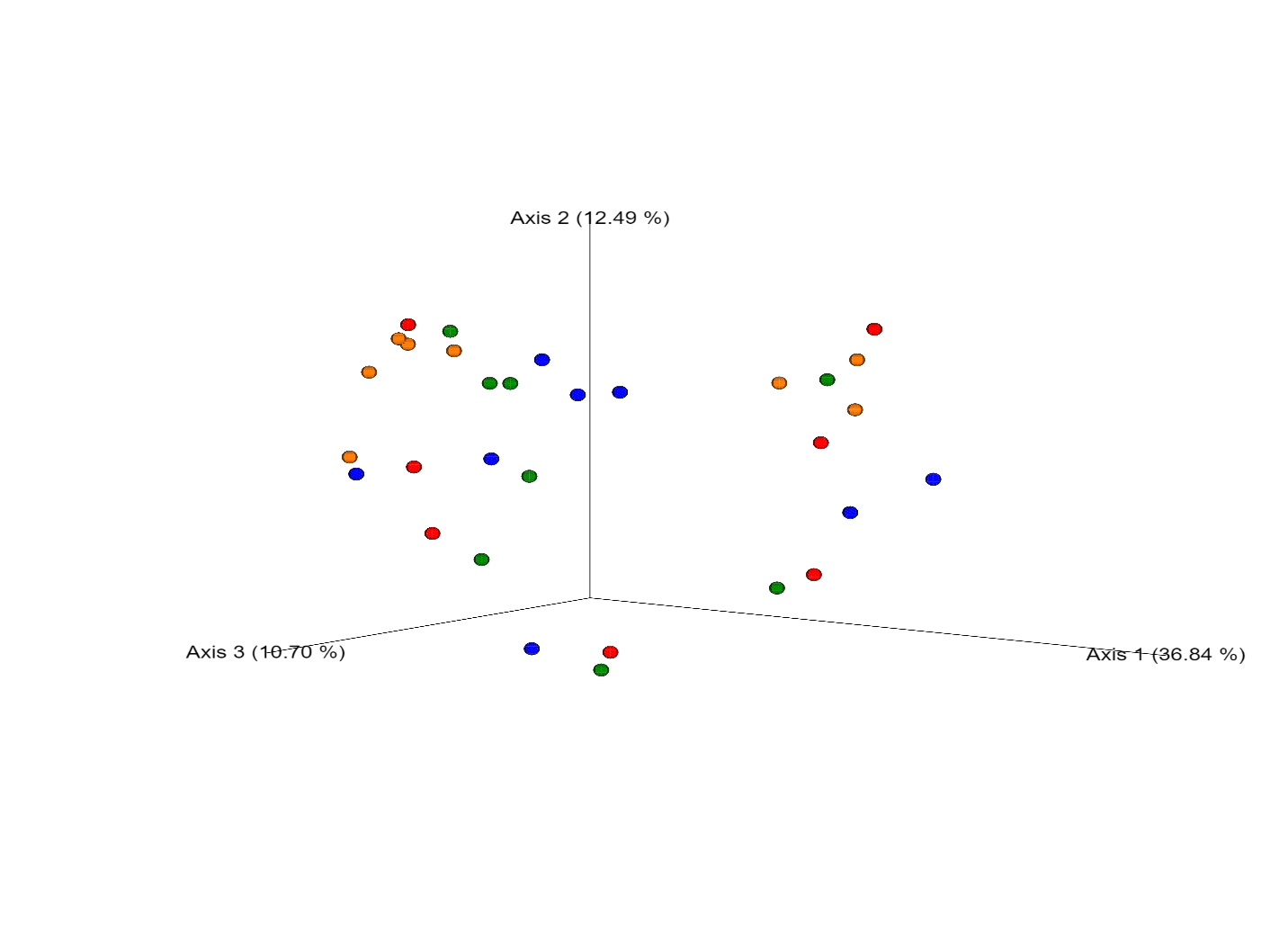


**(B)**

NC

PC

PPEO

PROB

**FIGURE S2**. Comparison of microbial population in broiler chickens fed various dietary supplements on d 42. (**A**) Shannon’s diversity index of each treatment group. Boxplots show the quartiles, median, and extremities of the values. (**B**) 3D PCoA plots based on weighted UniFrac distance matrix (bray-curtis). Each sphere represents a sample. Treatments include: negative control (NC): corn-soybean meal basal diet; positive control (PC): NC + 20 g virginiamycin (453 g Stafac^®^20)/ton diet; probiotic (PROB): NC + 227 g *B. licheniformis* spores/ton diet; probiotic/prebiotic/essential oil supplement (PPEO): NC + 453 g of a supplement containing *B. licheniformis* spores, mannan-oligosaccharides, β-glucans (1,3 and 1,6), capsaicin and curcuma/ton diet.
